# Comparison of pre-treatment methods and heavy density liquids to optimize microplastic extraction from natural marine sediments

**DOI:** 10.1038/s41598-022-19623-5

**Published:** 2022-09-14

**Authors:** Karin Mattsson, Elisabet Ekstrand, Maria Granberg, Martin Hassellöv, Kerstin Magnusson

**Affiliations:** 1 Department of Marine Sciences, Kristineberg Marine Research Station, University of Gothenburg, Fiskebäckskil, Sweden; 2 IVL Swedish Environmental Research Institute, Kristineberg Marine Research Station, 451 78 Fiskebäckskil, Sweden

**Keywords:** Environmental monitoring, Marine chemistry

## Abstract

The ubiquitous occurrence of anthropogenic particles, including microplastics in the marine environment, has, over the last years, gained worldwide attention. As a result, many methods have been developed to estimate the amount and type of microplastics in the marine environment. However, there are still no standardized protocols for how different marine matrices should be sampled or how to extract and identify these particles, making meaningful data comparison hard. Buoyant microplastics are influenced by winds and currents, and concentrations could hence be expected to be highly variable over time. However, since both high density and most of the initially buoyant microplastics are known to eventually sink and settle on the seafloor, marine sediments are proposed as a suitable matrix for microplastics monitoring. Several principles, apparatuses, and protocols for extracting microplastics from marine sediments have been presented, but extensive comparison of the different steps in the protocols using real environmental samples is lacking. Thus, in this study, different pre-treatment and subsequent density separation protocols for extraction of microplastics from replicate samples of marine sediment were compared. Two pre-treatment methods, one using inorganic chemicals (NaClO + KOH + Na_4_P_2_O_7_) and one using porcine pancreatic enzymes, as well as one with no pre-treatment of the sediment, were compared in combination with two commonly used high-density saline solutions used for density separation, sodium chloride (NaCl) and zinc chloride (ZnCl_2_). Both pre-treatment methods effectively removed organic matter, and both saline solutions extracted lighter plastic particles such as polyethylene (PE) and polypropylene (PP). The most efficient combination, chemical pre-treatment and density separation with ZnCl_2_, was found to extract > 15 times more particles (≥ 100 µm) from the sediment than other treatment combinations, which could largely be explained by the high presence and efficient extraction of PVC particles.

## Introduction

Anthropogenic microparticles, including microplastics, paint particles, tire and road wear particles, are particles in size range between 1 and 1000 μm^1^ that are intentionally or incidentally released into the environment by humans^2^. Land-based activities are the primary source of microplastic particles in the marine environment^3^. Primary microplastics, for intentional inclusion in products and applications, can be detected in the marine environment, as well as wear and friction particles, e.g., textile fibers or tire rubber, and the large class of microplastics from secondary fragmented macroplastics^1^. Plastic waste entering the ocean is expected to degrade and fragment due to physical, chemical, and biological processes such as UV radiation, wave action, and biodegradation^3^. Floating particles have been estimated to account for around 1% of the amount of plastic waste entering the oceans on a global scale^4^. Most of these plastic particles, in fact, will sooner or later sink and end up on the seafloor^5^, making sediment a sink for microplastics and thereby also a potential exposure route for these particles to marine organisms^6,7^. From a monitoring perspective, sampling surface water or the water column provides snap-shot information of microplastic pollution at a specific site^7^. However, plastic particles floating on the surface are highly influenced by wind, tides, and currents at the time of sampling^7^. In contrast, sediment microplastic monitoring can provide a more stable image of long-term accumulation, integrating the local or regional pollution levels on the time scales of years to decades^8,9^. This is also why monitoring most conventional hazardous substances is based on an analysis of sediments^10–14^.

There is no standard method for the isolation of microplastic particles from sediments. However, the most common approach is density separation with different saline solutions; based on the differences in density between plastic and sediment particles, light particles such as microplastics float while heavier particles sink. Commonly used high-density liquids are sodium chloride (NaCl) with a density of 1.2 g/cm^3^, zinc chloride (ZnCl_2_) with a density between 1.5 and 1.7 g/cm^3^, or sodium iodide (NaI) with a density between 1.6 and 1.8 g/cm^3^^15–18^. First, the sediment is mixed with the high-density liquid, agitated, and left to settle. The heavier mineral particles of the sediment sink to the bottom while the lighter particles, such as microplastics and natural organic particles, float to the surface due to their lower relative density. This density separation can be achieved in a beaker, separation funnel, or specially designed sediment extractors, e.g., the Munich Plastic Sediment Separator (MPSS)^19^. Next, the supernatant or buoyant fraction is filtered through one or a set of membranes with different mesh sizes, sorting the particles into size fractions to facilitate analysis. The membranes are then analyzed visually and/or spectroscopically^20^. Protocols can include pre-treatment of the sediments prior to extraction^21,22^, to digest natural organic matter to minimize interference during analysis and dissociate the matrix and thus make the microplastic particles more accessible for extraction. However, most protocols use post-extraction sample preparation based on elaborate treatment with enzymes or oxidative chemical reagents^16,23–25^ to facilitate subsequent analysis using spectroscopy^26–28^. The selected treatment should be efficient in removing interfering natural particulates while not chemically or physically damaging the plastics^29–31^.

In monitoring recommendations^10,14,32^, a subset of visually inspected microplastics representative of the sample is often proposed to be validated by spectroscopic identification. However, it is not expressed which particles should be selected other than that they should be representative of the particles found within the matrix. The selection itself may present a potential bias in the microplastic analysis of environmental samples. Visual analysis, which includes both visual and tactile identification and sometimes particle heating, i.e., poking the particle with a hot needle, has been criticized because of the risk of misidentification, resulting in false negative and false positive identification of plastic particles^20,33–35^. On the other hand, spectroscopy builds on chemical spectra interpretation^36^, and can provide spectroscopic clues on microplastic identification, including specific polymer composition^2^. However, computer-based database match identification can be prone to mismatch^37,38^, and operator bias may also lead to bias in spectroscopic identification^39^. Furthermore, spectroscopically identifying all particles in a sample manually is time-consuming, and agreement on the criteria for spectral matching has not been decided. Automated higher throughput methods are being developed and used, allowing all particles to be probed spectroscopically using a fixed computerized instruction considered to minimize operator bias^39,40^. However, from a monitoring perspective, the automated methods are not yet fully established and proven in terms of time and cost-efficiency^36,40^.

The main aim of this study was to evaluate the efficiency of different pre-treatments, density separation solutions, and their combinations for the extraction of microplastics from natural muddy marine sediments. Moreover, to evaluate the mutual accuracy of identification methods, visual approach followed by chemical identification. Thus, we compared three conditions, a non-pre-treatment of the sediment, enzymatic treatment, and inorganic chemical treatment in combination with two density separation solutions, NaCl and ZnCl_2_ (3 treatments and 2 density separation solutions, $n=3\times 2=6$^41^).

## Materials and methods

### Sediment sampling

Ten sediment cores were collected at a station in Askeröfjorden, N58°5′21′′ E11°48′6″ (center), outside Stenungsund, Swedish west coast, on 24th October 2018, using a Gemini corer. The sediment was characterized following recommendations from the Swedish Geotechnical Society^42^ as a sandy, silty gyttja-clay, bioturbation and benthic macrofauna species were identified, with the highest abundance of the species *Amphiura* spp. and *Arctica islandica*. The top 2 cm from all cores were pooled, homogenized in a 10 L stainless steel pot with a stainless-steel spoon, and transferred to 15 glass containers with glass lids, each with a volume of 200 mL (approximately 314 g) (Table 1). All samples were stored at 8 °C until further analysis.

**Table 1:** Identified microparticles in blank samples and sediment samples treated with inorganic chemicals, enzymes and with no treatment for both density separation solutions, ZnCl_2_ and NaCl.

Density solution	Pre-treatment	Mean number of identified microparticles > 300 μm per sample	Mean number of identified microparticles 300–100 μm per sample^a^	Mean dry weight (g)
ZnCl_2_	None	30	120	268
	Inorganic chemicals	14	1058	380
	Enzymatic	29	250	310
ZnCl_2_	Blank none	1	7	314
	Blank inorganic chemicals	0	6	314
	Blank Enzymatic	0	5	314
NaCl	None	14	149	324
	Inorganic chemicals	3	259	319
	Enzymatic	13	254	300
NaCl	Blank none	7	5	314
	Blank inorganic chemicals	3	3	314
	Blank enzymatic	4	9	314

^a^Includes anthropogenic particles identified from the supernatant of washed samples.

### Sediment treatment

Sediment samples were weighed before a subsample of 5 mL was retrieved and dried at 105 °C for 24 h for analysis of water content. The sediment samples (n = 15) were divided into three groups, one group was treated with inorganic chemicals (n = 4), one with enzymes (n = 5), and the third remained untreated (n = 6). The treatment with inorganic chemicals is based on a protocol developed by Strand and Tairova 2016^22^ but slightly modified as it consisted of a mixture of 0.67 mol/L NaClO, 0.45 mol/L KOH, and 0.022 mol/L Na_4_P_2_O_7_. Approximately 400 mL of the chemical treatment (twice the volume of the sediment) was added to the glass containers with glass lids with the sediment. The samples were incubated at room temperature for 1 h on an oscillating stirring table at 160 rpm. An additional rinsing step was required after the chemical treatment to lower the pH and avoid precipitation of zinc hydroxides. Subsequently, the sediment was washed by adding Milli-Q water followed by vigorous shaking and centrifugation for 30 min at 1000 rpm. This washing procedure was repeated three times. The supernatant was removed between repetitions, and new Milli-Q water was added. The supernatant was filtered through a 50 μm filter, which was kept for further analysis. After centrifugation, the sediments were transferred back to the glass containers. Porcine pancreatic enzymes were used for the enzymatic digestion of organic matter^43^. A buffered enzyme working solution was prepared by dissolving one capsule of the pharmaceutical enzyme (Creon 40 000, Abbott Laboratories GmbH, Germany, Mylan) per 10 mL of Tris hydrochloride solution (Trizma, pH 8.0, 1 M, 0.2 μm filtered, Sigma-Aldrich, T3038, USA). Complete dissolution was achieved through gentle warming (30 °C) and shaking at 125 rpm for 30 min on a heated incubation shaker (New Brunswick Scientific, Innova 40). Ten mL of the working solution was added per g wet weight (WW) of sediment in a prewashed glass jar. The sediment-enzyme solution was mixed, and the pH was assessed using a pH indicator stick. The pH was adjusted to 8.0 by adding more Tris hydrochloride solution if necessary. The filled glass containers were lidded and placed on vigorous shaking (150 rpm) at 37.5 °C overnight on the incubation shaker.

The level of degradation was quantified by TOC analysis using an elemental analyzer coupled to an isotope ratio mass spectrometer (20–22, Secron Ltd., Crewe, UK) before and after degradation treatment.

### Density separation and filtration

**Figure 1:**
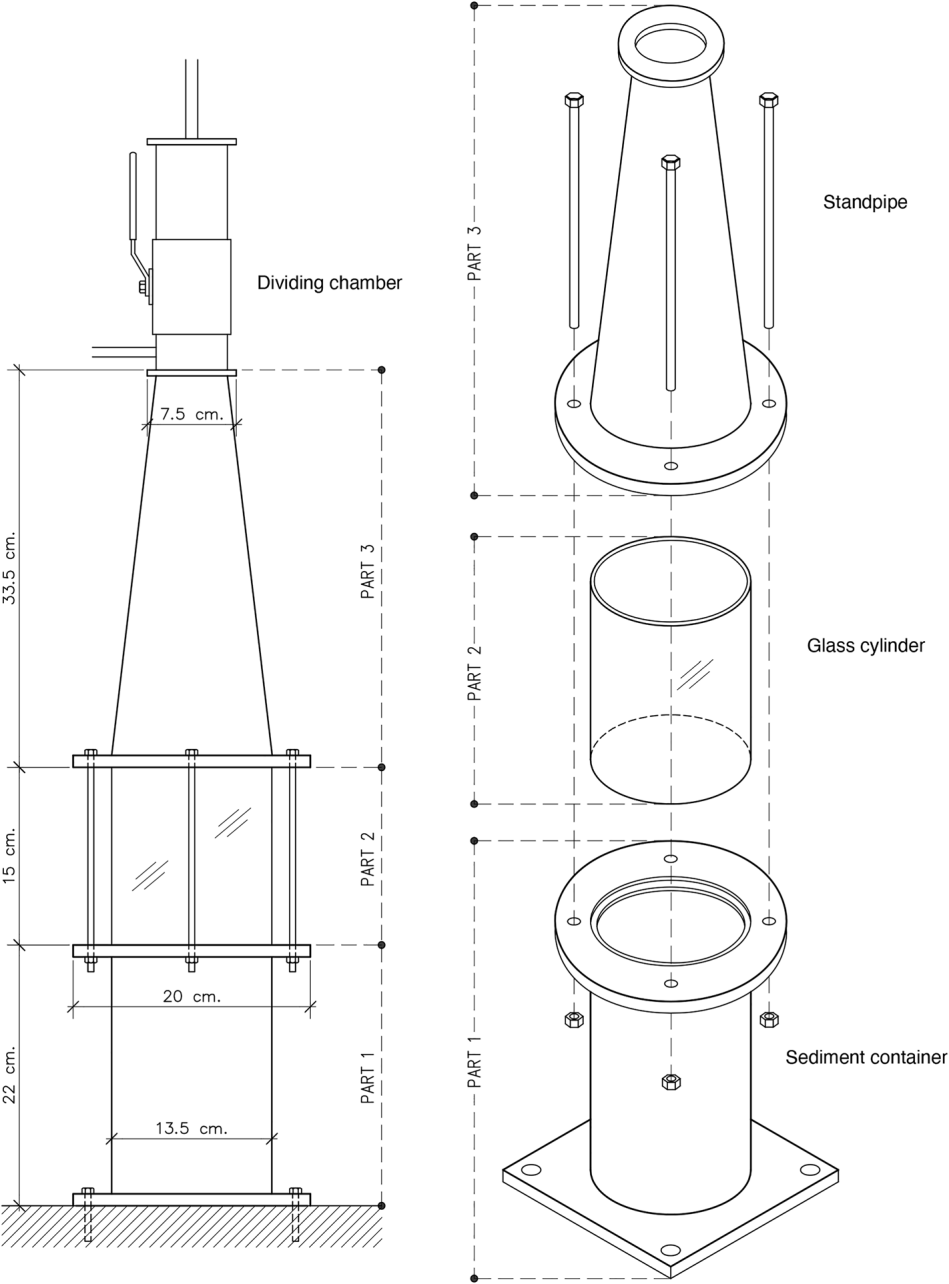
The Kristineberg microplastic sediment separator (KMSS) designed after the Munich plastic separator (Imhof et al., 2012). Part 1, sediment container with a rotor and a bottom valve, part 2, glass cylinder, part 3 standpipe, and top part dividing chamber with ball valve and filter holder.

To compare the efficacy of two different widely used density separation solutions, NaCl (density 1.2 g/cm^3^) and ZnCl_2_ (1.8 g/cm^3^), the pre-treated samples were divided into two groups further processed with ZnCl_2_ (n = 7) or NaCl (n = 8). The density separation was performed using the Kristineberg Microplastic Sediment Separator (KMSS). This separator has been designed in-house with inspiration from the Munich Plastic Separator^19^, but is smaller, both in height and width, with a steeper incline on the standpipe with a glass cylinder above the sediment container to monitor the sedimentation (Fig. 1).

After pre-treatment, the sediment samples were moved to the bottom sediment container of the KMSS, the standpipe part of the tower was mounted, and the rotor, positioned in the bottom of the tower, was turned on. The saline solution was introduced through the bottom valve and filled to 85% of the tower's volume. The rotor was turned off three hours later, and the sediment was allowed to settle. After 12 h of settling, the dividing chamber was mounted on top of the standpipe, and the tower was filled with the density separation fluid, either ZnCl_2_ or NaCl. When filled, the ball valve was closed, the liquid level lowered, and the dividing chamber was removed. Next, the dividing chamber was turned upside down, and the solution was filtrated. The sediment exposed to the pre-treatment with inorganic chemicals and separated with ZnCl_2_ was filtrated through a 300 μm polyamide (PA) mesh and the remaining top solution was collected, and a second separation with ZnCl_2_ in a glass beaker was performed due to high mineral content. After the second-density separation (agitation and left to settle for 24 h), the solution was filtered through a PA filter with mesh sizes 100 μm. For the enzymatic and none treated samples as well as all samples extracted with NaCl, only one separation was performed since fewer mineral particles were extracted compared to pre-treated with inorganic chemicals and separation with ZnCl_2_. The solution was filtrated through two PA filters with mesh sizes 300 μm, and 100 μm. After filtration, the filters were rinsed with Milli-Q water to remove salt crystals.

### QA/QC

All equipment used during sampling was cleaned and rinsed in the laboratory with Milli-Q water before drying in a plastic-free flume hood. For the storage of sediment samples, glass containers with glass lids were used, and a stainless-steel pot and a stainless-steel spoon were used for homogenization of the sediment. All samples were stored in their glass bottles until processed in the lab. All equipment and lab surfaces were cleaned before laboratory work, and only cotton lab coats and clothes were worn. The ZnCl_2_ was filtrated through three membranes 10 μm, 5 μm, and 1 μm. The NaCl was filtrated through 10 μm.

Six blank samples without sediment were processed, one for each combination of pre-treatment and density separation solution. All blanks were treated as their corresponding sediment sample from cleaning the bottles before sampling, in the field, during storage, treatment, separation, and analysis.

### Analysis

The analysis workflows followed the current consensus guidelines, either published or available as working drafts from regional seas conventions and the European Commission^10,14,32^. All filters were first visually inspected with a stereomicroscope (Leica M205 C 80–160× Wetzlar, Germany). Subsequently, all particles that were visually identified as suspected anthropogenic following Karlsson et al. 2020^44^ were transferred with a tweezer to different aluminum-oxide filters with a pore size of 200 nm (Whatman Anodisk 25). One filter for each treatment and size fraction, i.e., the particles collected on the 300 μm mesh were moved to one aluminum-oxide filter, and the particles from the 100 μm were moved to another. The particles collected from the centrifugation solution were also transferred to one filter. Two filters were used if there were more suspected particles than what could fit on one Anodisk filter. The entire area of the Anodisk filters with particles was imaged with a light microscope (Zeiss, AxioImager). All particles (collected on the 300 μm filters, 100 μm filters, and from the centrifugation solution, 50 μm) were characterized according to their visual appearance, following Karlsson et al. 2020^44^ were their 2D shape, 3D shape, solidity, color, and classical visual identification were noted. Subsequently, all particles from the 300 μm filter and all particles on eight randomly chosen 100 μm samples were chemically identified with Raman microscopy (Witec, alpha 300R) using a 532 nm laser and 600 g/mm grating. The laser power was selected based on the polymer, signal intensity, size of the particles, and magnification. Spectra were measured in a wavenumber range of 200 to 3500 cm^−1^ and were compared to our in-house library for identification (HQI minimum 75, majorities above 80). The library consists of spectra we have obtained from known plastic particles, including weathered particles, and spectra from the RUFF^45^ and ST Japan spectral databases. For comparison, 300 µm filters from two randomly chosen samples were also chemically identified with Fourier-transform infrared spectroscopy, FTIR (Thermo Scientific Nicolet iN10) using transmission mode (256 scans, resolution 4 cm^−1^, spectral range 4000–675 cm^−1^, detector cooled by liquid nitrogen and correlated against 256 background scans). The particles in the supernatant from the centrifugation solution (all inorganic pre-treatments and one of the enzymes pre-treated) were first visually characterized before being analyzed with Raman microscopy. The remaining particles on the PA filter (300 μm, 100 μm, and 50 μm from centrifugation solution) from two random samples, i.e., the particles that were not visually identified as anthropogenic particles, were analyzed with Raman microscopy to identify false negatives.

## Results and discussion

### Particle identification, sizes, and concentrations

**Figure 2:**
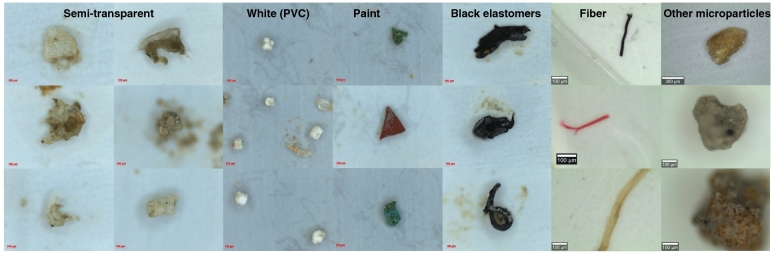
Categories of typical particles, semi-transparent plastic particles, white (PVC particles), paint particles, black elastomers, synthetic fibers, and other anthropogenic microparticles.

**Figure 3:**
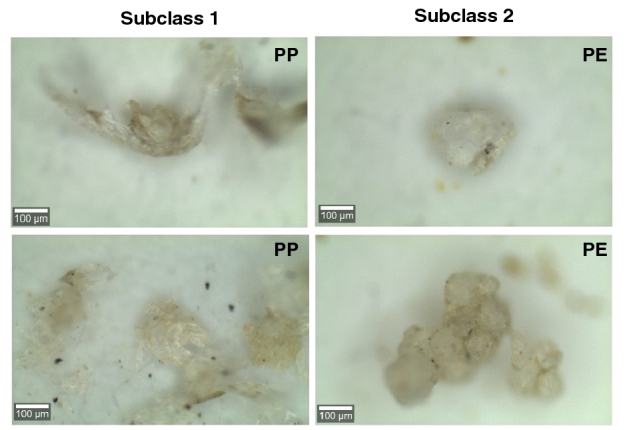
Semi-transparent plastic particles in subclass 1 (PP) and subclass 2 (PE).

**Figure 4:**
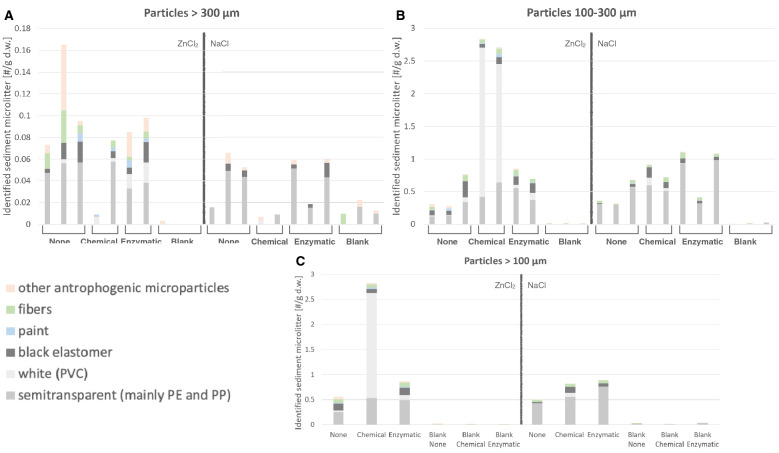
Particle concentration of anthropogenic microparticles for particles (**A**) larger than 300 μm, (**B**) between 100 and 300 μm, and (**C**) larger than 100 μm.

The anthropogenic particles extracted from the sediment were first categorized using a stereomicroscope according to their visual and tactile appearance^44,46^. The categories used were semi-transparent microplastics, white microplastics, black firm elastomer, paint particles, synthetic fibers, and other anthropogenic microparticles (Fig. 2). On the 300 μm filter, most of the plastic particles were semi-transparent (59% of 277 particles) and visually divided into three subclasses based on apparent differences in morphology. Subclass 1 had a stripe pattern, subclass 2 was built up by spherical patterns, and subclass 3 was the semi-transparent particles that had neither stripes nor a spherical pattern. All particles within visual subclass 1 were confirmed to be PE (n = 106), and all particles within subclass 2 were confirmed to be PP (n = 15) (Fig. 3). In subclass 3, polymers such as PS and PMMA were identified. This implies that, in this case, visual identification could be used to separate the semi-transparent particles into subclasses with different polymer compositions. The specific surface patterns of the semi-transparent PP and PE particles indicate that they both derive from distinct sources. In total, 66% of the semi-transparent particles on the 300 μm filter were identified as PE and 10% as PP (Fig. 4A). As expected, more anthropogenic particles were collected on the 100 μm filter, in total, 4329 particles (Fig. 4B, Table 1). On the 100 µm filters, there were two main categories of plastic particles, semi-transparent and white, representing 46% and 38% of the total number, respectively. All spectroscopy analyzed particles (n = 335) were confirmed to be PE, PP, and PVC, as identified visually. From the supernatant of the centrifugation samples, the majority of the particles (64%) were categorized as semi-transparent and spectroscopically identified as PE (n = 47) and PP (n = 9).

The highest concentration, 2.82 microplastic particles per g dry weight (DW) of sediment, was found in the samples exposed to the inorganic chemical pre-treatment and extracted with ZnCl_2_. Most of these particles (74%) were identified as PVC (Fig. 4). The number of PVC particles was distinctly higher in the combination of inorganic chemical pre-treatment and ZnCl_2_ compared to the other treatments, 1.81 to 2.29 PVC particles/g DW sediment compared to 0–0.12 PVC particles/g DW sediment (Fig. 4C).

The concentration of semi-transparent plastic particles, i.e., mainly PP and PE particles, ranged from 0.25 to 0.76 particles per g DW sediment. The highest concentrations of semi-transparent microplastics were found in samples pre-treated with enzymes and extracted with NaCl. The lowest concentration was identified in samples which had not been treated to remove labile organic matter, independent of density separation solution. However, a certain difference in particle distribution could be expected, regardless of treatment or density separation solution, since it is not possible to obtain sediment samples that are perfectly identical.

### Blank samples

From the blanks, only between 5 and 13 particles per sample were extracted, including only the categories semi-transparent microplastics, synthetic fibers, and other anthropogenic microparticles. In the blank samples extracted with ZnCl_2_, most anthropogenic particles belonged to the category of other anthropogenic microparticles, while in those extracted with NaCl, semi-transparent plastic particles dominated, followed by synthetic fibers and other anthropogenic microparticles. White microplastic particles were not extracted from any of the blank samples. The blank sample with the highest number of extracted microplastics was from the pre-treatment with enzymes and extracted with NaCl, where 13 particles were identified, and 10 of these were categorized as semi-transparent microplastics, subclass 3. All particles visually identified as anthropogenic in the blank samples are shown in Fig. 4.

Comparing the extracted particles from the blank with the particles in the sediment samples show a significant difference in plastic concentrations between the blanks and the sediment samples, p < 0.001 (2 sample T-test) (Fig. 4).

### False negatives using visual identification

There is a scientific concern regarding the visual identification of microplastic particles because of the risk of bias between operators and the risk of both false positive and false negative identifications^20,33–35^. The particles remaining on the PA membranes after removal of all particles identified with stereomicroscopy as being composed of plastic, i.e., all particles visually considered to be non-anthropogenic, were analyzed with Raman microscopy. On the membranes from the pre-treatment with enzymes and extracted with NaCl, 3 particles on the 300 μm and 11 particles on the 100 μm were identified as plastics, representing 2% and 13%, respectively. On membranes from the pre-treatment with inorganic chemicals and extracted with ZnCl_2_, there were 5 particles remaining and later identified as plastic on each of the membranes, representing 1% of the total amount of particles. This shows that, at least in the present study, a skilled operator was able to identify plastic particles down to 100 μm with few false negatives, using visual and tactile identification techniques.

### Effects of pre-treatment steps

The two pre-treatments (inorganic chemicals and enzymatic) that served to reduce the organic matters stickiness in the sample, thus breaking microplastics-matrix adhesion, was functionally evaluated with TOC analysis. The inorganic chemical pre-treatment had a TOC content of 1.55%, whereas the untreated sample had 2.89%. On the other hand, the enzymatic treatment showed a higher TOC level, 4.02%, most likely since enzymes add carbons to the sample. However, from the recovery rates, it is clear that for particles > 300 μm, the identified concentrations of particles from the different pre-treatment methods were similar to the untreated samples showing that if only > 300 μm particles are of interest, a pre-treatment is not necessary. However, for smaller lighter particles (PVC excluded) between 100 and 300 μm, a significant difference in concentrations between pre-treated and untreated samples was found (p = 0.00116). Moreover, there is a significant difference (p < 0.001) in plastic concentrations found in the sediment samples pre-treated with inorganic chemicals and extracted with ZnCl_2_, mainly due to this combination's high extraction of PVC particles.

Our results are in agreement with Enders et al. (2017), who tested KOH in combination with NaClO for digestion of fish stomach and showed that this treatment effectively digested the tissue. Moreover, they tested if the treatment affected the Raman spectra of 12 common polymers and only found a low peak alternation for acrylonitrile butadiene styrene (ABS) particles; however, they were still able to identify this polymer^30^. The enzymatic treatment has also been shown to effectively digest tissue^43^ without harming common plastics^47^.

### Density separation solution

There was a higher extraction efficiency of plastic particles with ZnCl_2_ than with NaCl. Heavier polymers such as PVC were recovered with ZnCl_2_ (Fig. 4) but not with NaCl, as is expected based on the lower density of the NaCl saline solution. From a monitoring perspective, NaCl is an attractive alternative since it is non-toxic and has a lower cost than ZnCl_2_ (technical grade ZnCl_2_ is ~ 4 euros per kg). However, NaCl is not recommended when aiming to target particles with a higher density than 1.2 g/cm^3^, e.g., Polyethylene terephthalate (PET) or PVC. For a comprehensive polymer extraction, a higher density solution (> 1.5 g/cm^3^) should be recommended since it can extract the majority of polymers also including PET and PVC. A density of at least 1.6 g/cm^3^ for saline density separation solutions was recently recommended for Arctic microplastic monitoring programs^14^.

## Conclusions

Higher concentrations of microplastic particles were identified in samples pre-treated with either inorganic chemicals or enzymes to remove labile organic matter, as compared to non-pre-treated samples, showing that a pre-treatment step improves the extraction of microplastics. However, if only particles > 300 μm are considered, the pre-treatment step was not necessary. Both density separation solutions, NaCl and ZnCl_2,_ successfully extracted lighter microplastic particles such as PE and PP. In addition, the combination of the inorganic pre-treatment with ZnCl_2_ successfully extracted PVC particles. From a monitoring perspective, our results are in agreement with the Arctic Monitoring and Assessment Program (AMAP)^14^, which recommends a density of at least 1.6 g/cm^3^ to achieve a comprehensive polymer extraction which also includes PET and PVC particles.

## Data Availability

The datasets used and analyzed during the current study are available from the corresponding author on reasonable requests.

## Abbreviations

ABS: Acrylonitrile butadiene styrene
AMAP: Arctic Monitoring and Assessment Program
DW: Dry weight
FTIR: Fourier-transform infrared spectroscopy
KMSS: Kristineberg microplastic sediment separator
MPSS: Munich plastic sediment separator
PA: Polyamides
PE: Polyethylene
PET: Polyethylene terephthalate
PMMA: Poly(methyl methacrylate)
PP: Polypropylene
PS: Polystyrene
PVC: Polyvinyl chloride
WW: Wet weight
